# An ultrasound model to calculate the brain blood outflow through collateral vessels: a pilot study

**DOI:** 10.1186/1471-2377-13-81

**Published:** 2013-07-11

**Authors:** Paolo Zamboni, Francesco Sisini, Erica Menegatti, Angelo Taibi, Anna Maria Malagoni, Sandra Morovic, Mauro Gambaccini

**Affiliations:** 1 Vascular Diseases Center, University of Ferrara, Via Aldo Moro 8, 44124, Cona, (FE), Italy; 2 Department of Physics, University of Ferrara, Via Saragat 1, 44122 Ferrara, Italy; 3 Aviva Medical Centre, Nemetova 2 10 000, Zagreb, Croatia

**Keywords:** Chronic cerebro-spinal venous insufficiency, CCSVI, Internal jugular vein, IJV, Echo colour doppler, Model, Ultrasound, Haemodynamics, Cerebral outflow

## Abstract

**Background:**

The quantification of the flow returning from the head through the cervical veins and the collaterals of the internal jugular vein (IJV), is becoming of prominent interest in clinical practice. We developed a novel model to calculate the cerebral venous return, normalized to the arterial inflow, in the different segments of the IJV.

**Methods:**

We assessed, by established Echo Colour Doppler (ECD) methodology, the head inflow (HBinF) defined as the sum of common carotids and vertebral arteries, as well as the cerebral flow (CBF) defined as the sum of internal carotid and vertebral arteries. We also assessed the head outflow (HBoutF) defined as the sum of the measurements at the junction of the IJV and the vertebral veins. In addition, we also calculated the collateral flow index (CFI) by estimating the flow which re-enters directly into the superior vena cava as the amount of blood extrapolated by the difference between the HBinF and the HBoutF. We preliminarily tested the model by comparing ten healthy controls (HC) with ten patients affected by chronic cerebral spinal venous insufficiency (CCSVI), a condition characterized by some blockages in the IJV which are bypassed by collateral circulation.

**Results:**

In HC the HBinF was 956+-105ml/min, whereas the HBoutF was > 90% of the HBinF, leading to a final CFI value of 1%. The last result shows that a very small amount of blood is drained by the collaterals. In upright we confirmed a reduction of the outflow through the IJV which increased CFI to 9%. When we applied the model to CCSVI, the HBinF was not significantly different from controls. In supine, the flow of CCSVI patients in the IJV junction was significantly lower (p < 0.001) while the correspondent CFI value significantly increased (61%, p < 0.0002).

**Conclusions:**

Our preliminary application of the novel model in the clinical setting suggests the pivotal role of the collateral network in draining the blood into the superior vena cava under CCSVI condition.

## Background

There is general agreement in considering the internal jugular veins (IJVs) as the major route of cerebral outflow in the supine position, and the vertebral veins (VVs) as the major route of brain drainage in upright [1-4]. In a recent consensus, the IJV was subdivided into 3 segments: the segment J3 or higher, which is anatomically located at the carotid bifurcation and the mandibular angle; the middle segment or J2, related to the ipsilateral thyroid lobe; finally, the lower end or J1, corresponding to the confluence with the brachio-cephalic vein trunk [5].

In a recent paper we have shown that the flow tends physiologically to grow in volume from J3 to J1, both in basal conditions and under standardized conditions of activation of the thoracic pump [6]. The main question to be answered is why the IJV flow is increased from the skull to the chest. Our hypothesis is to consider the possibility of blood re-entry from jugular collaterals into the main trunk (in the following we will use the term re-entry to indicate when a collateral channel is flow tributary of the major truncal pathway). Furthermore, the increase of IJV flow along the extra-cranial segment could be related to the re-entry volume through collateral vessels draining not only the blood of face and neck soft tissue, but also a rate coming from the brain through extra-intra-cranial anastomosis. In fact, there is a never assessed quota of the head inflow that is conveyed into the IJV more caudally with respect to the J3 position, through intra- and extra-cranial anastomosis. We are aware of anatomical presence of intra- and extra-cranial connection [7] but their physiological contribution to brain circulation is completely unknown. To this aim we have developed an haemodynamic model which describes quantitatively the neck pathway of the cerebral venous return, normalized with respect to the arterial inflow. Flow parameters have been measured by means of established echo-colour Doppler (ECD) methodology. Finally, in the second phase of the research, we have performed preliminary measurements in normal subjects and in patients affected by Chronic Cerebrospinal Venous Insufficiency (CCSVI) [5].

## Methods

### First phase of the study

Total of eleven healthy volunteers were screened for CCSVI absence by means of established ECD criteria [5]. One out of eleven screened subjects presented with >2 ECD criteria positive for CCSVI and, consequently, was excluded from the healthy controls. Ten out of eleven entered the study (age ranging from 23 to 42 y.o., male:female ratio 3:2). This prospective study was in accordance with *Ethical Standards of the Committee on Human Experimentation* of the University of Ferrara. All the study participants were non-invasively investigated by means of ultrasonic scanning with an ECD machine (ESAOTE My-Lab 70, Genoa, Italy) at the same condition of room temperature (23° Celsius) and with all participants off of drugs influencing the venous tone. Measurements were all performed in the morning hours following recommendation to drink 500 ml after the wake, in order to have comparable conditions of hydration [5].

### Protocol of ECD measurement

#### *Subject positioning and condition of measurement*

Each experiment was performed with the subject placed on a tilting chair in both supine and upright positions while breathing normally, by starting the examination in supine position. After changing position, an adaptation period of at least 2 minutes was allowed before any further measurement.

The examiner carefully observed the inclination of the patient’s neck and provided appropriate neck support to avoid neck flexion, hyperextension or rotation to the left or right, which could potentially compress the neck veins and consequently affect measurements.

We used a thick layer of ultrasonic gel as well as recommended maneuvers in order to reduce excessive pressure on the patient’s neck that may change the shape and size of the IJV [5].

#### *Evaluation of Doppler venous haemodynamics*

Total inflow and outflow volume per unit of time, namely the flow Q, was measured in both supine and sitting position for each volunteer.

Inflow has been measured at common carotid artery (CCA) just below the bifurcation, and at the proximal segment of both internal carotid (ICA), and external carotid (ECA) artery.

The vertebral artery (VA) was evaluated at V2 level for reproducibility reasons [5]. In addition, outflow was measured in sequence at J2, J3, J1 level of the IJVs and at C4-C5 level of the VVs [5].

Two different approaches were used to calculate inflow and outflow, depending on the different shapes of the cervical arteries and veins. The investigated arteries have almost circular cross sectional area (CSA), so the CSA in this case was calculated using the diameter measured in longitudinal aspect of the B-mode imaging. Therefore, the Doppler sample volume was placed in the artery with the sample aperture corresponding to the lumen, in order to perform flow measurements by means of uniform insonation techniques [8-10].

On the contrary, since the IJV exhibits an elliptical shape, the CSA and major axis were assessed in the transversal aspect of the B-mode imaging by manually tracking the boundaries of the lumen.

The mean velocity of the blood in the veins has been calculated in accordance with the assumed profile technique, namely by sampling such velocity at the point where its value is highest. To do this, a smaller sample volume of 0.5 mm has been adopted for three reasons: 1) the small sample volume assures that the Doppler angle is constant over the whole sample volume, 2) the use of constant sample volume simplifies very much the on-line work of the Doppler operator and thus assuring a more accurate measurement 3) the use of small sample volume minimizes the vessel wall artifacts [8-12].

In the segment J1 of the IJVs, when we observed either an absent or a turbulent flow, we calculated the velocity profile by finely sampling in five different positions from wall to wall.

For both arteries and veins, the duration of the acquired Doppler spectrum was 4 seconds. For the arteries we considered three cardiac cycles while for the veins one respiratory cycle.

#### *Off-line assessment of Doppler haemodynamics*

We carefully acquired images and traces as above described, trying to improve as much as we could the reliability of the Doppler assessment and of the variables determined by the operator (Angle, PRF, etc.). In particular, the actual Doppler angle has been always carefully checked off-line and the contour of the jugular cross section has been determined by observing the movement of the vessel wall during the respiratory cycle.

Actual measurements were carried out by EM while during the acquisition EM and FS agreed on the Doppler technique regarding angle, position of the SV, etc. Since measurements of both inflow and outflow took a long time, calculation of the haemodynamic parameters was performed off-line by using the stored images, in order to shorten the examination time and to avoid possible physiological changes.

The flow Q was calculated as *Q* = *TAV* × *CSA*, where TAV is the time average velocity of the blood when considering one respiratory cycle for the veins and three cardiac cycles for the arteries. TAV was calculated as $\mathrm{TAV}=\mathrm{TA}V_{p}\times \frac{1}{\eta }$, where TAV_p_ corresponds to the average velocity measured on the peak of the trace and η is the velocity factor [13] calculated following Vergara [14] and using the Womersley number [15].

#### *Refinement of Doppler haemodynamics assessment*

Off-line calculation permitted also to improve the accuracy of the derived parameters. Post-processing allowed us to record the minimum and maximum CSA during respiratory cycle by manual tracing. After that, the venous flow Q was determined by calculating the mean value of the CSA.

A second parameter needing accurate post-processing verification is the angle of the Doppler beam for the vessels under measurement (Doppler angle). Such parameter and the uncertainty of the operator in placing it usually affect the TAV assessment. In our off-line processing we managed to estimate the uncertainty of TAV measurements as described in [16]:

(1) $$\delta \mathrm{TAV}=\mathrm{TAV}\times \left(\frac{\cos\left(\theta \right)}{\cos\left(\theta +\epsilon \right)}-1\right)$$

where θ is the incident angle of the Doppler beam, and ϵ is the uncertainty of the operator. The uncertainty of the flow is given by: *δQ* = *δTAV* × *CSA*.

#### *Parameters of head and brain circulation*

All the measurements for the above mentioned arteries and veins have been taken on both right and left sides. In particular, the carotids have been measured in the CCA segment, in the ICA segment and in the ECA segment. In order to minimize the experimental error we assume that the total head blood inflow (HBinF) is:

(2) $$\mathrm{HBinF}=\frac{\mathrm{CCAs}+\left(\mathrm{ICAs}+\mathrm{ECAs}\right)}{2}+\mathrm{VAs}$$

whereas the cerebral blood flow (CBF) was roughly assumed to be the sum of ICAs and VAs contribution and then calculated as the sum of ICAs and VAs flows [17]. The cerebral venous outflow (CVO) was calculated as the sum of the flow measured at level J3 of the IJVs and the flow measured in the VVs. The total head blood outflow (HBoutF) was calculated as the flow of both left and right IJVs at J1 plus the VVs flows.

#### *Model of neck veins*

**Figure 1:**
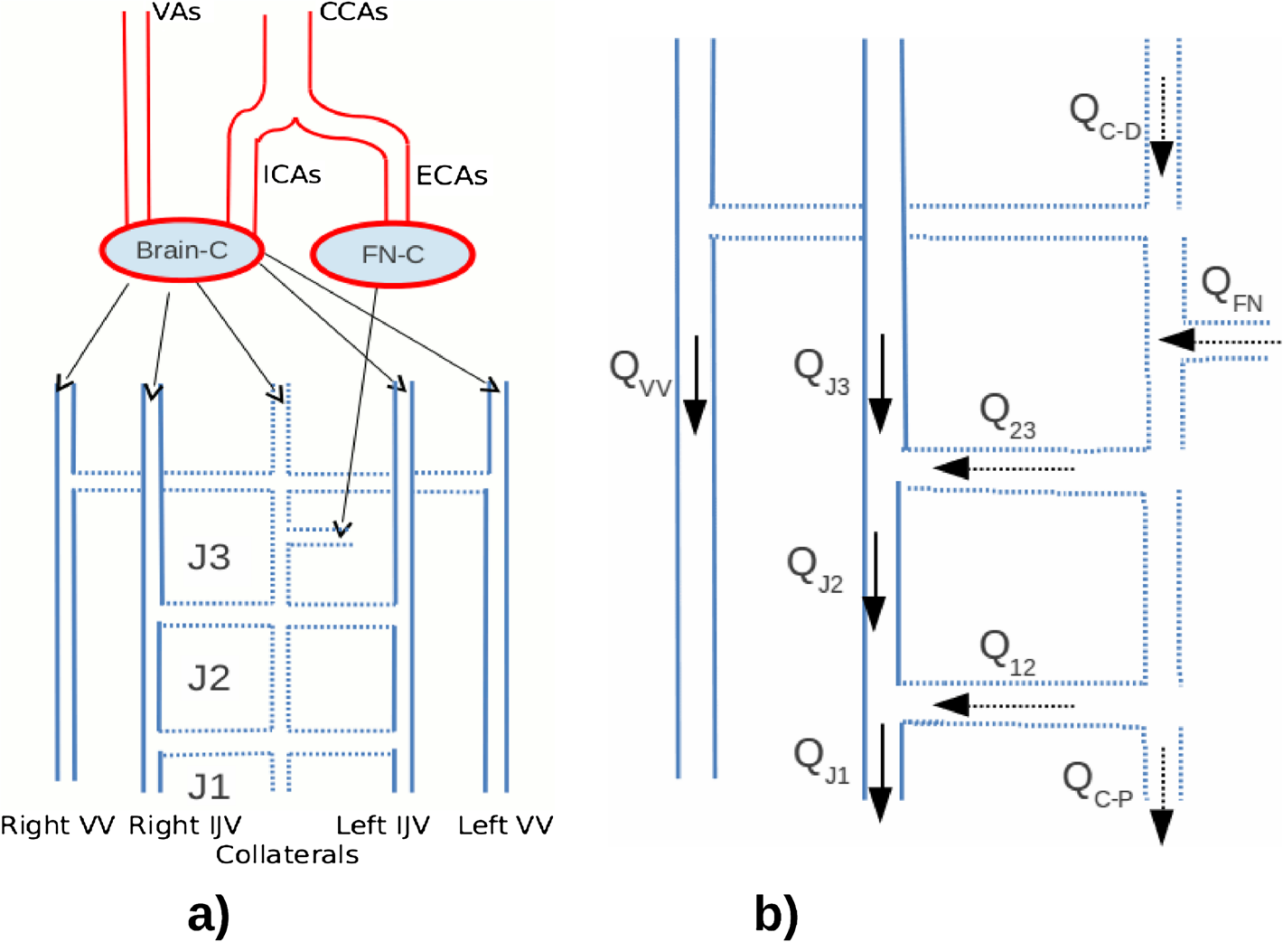
**Model of the neck pathway concerning the cerebral venous return. a)** Red tubes represent inflow arteries vessels while blue tubes represent outflow venous vessels. The dashed line are used to represents the collateral venous network. **b)** Representation of the flow in the vessel of right network of the neck. The direction of the arrows indicate the physiological direction of the flow.

In order to analyze the results we propose a haemodynamic model (Figure 1) which includes the neck pathways of the cerebral venous return.

As shown in Figure 1a the red tubes represent the inflow vessels (CCAs, ICAs, ECAs and VAs) while the blue ones represent the outflow vessels (VVs e IJVs). The CCA is divided in ICA and ECA. VAs and ICAs enter the brain compartment (Brain-C) and then the flow is normally drained by IJVs, VVs and collateral veins which are represented in the model by blue coloured tubes. The ECAs enter the facial and neck compartment (FN-C) and then are mainly drained by the collateral veins of the face and neck.

Vessel drawn with a continuous line are those evaluated by ECD in this study (CCAs, ICAs, ECAs, IJVs e VVs) while the ones with a dotted line are collateral veins which have been inserted in our model to account for the variations of the jugular flow.

In Figure 1b, flow directions are represented by a continuous arrow: Q_J3_, Q_J2_ and Q_J1_ are the measured flows in J3, J2 and J1 respectively, while Q_vv_ is the measured flow in VV. Figure 1b also shows the collateral flows by means of a dotted arrow.

From top to bottom we now describe in detail the collateral flows of Figure 1b.

Q_C-D_ (Collateral-Distal) is the brain outflow which goes directly into the collateral network:

(3) $$Q_{C-D}=\mathrm{CBF}-\mathrm{CVO}$$

Q_FN_, is the flow coming from the facial-neck compartment and going again into the collateral network:

(4) $$Q_{\mathrm{FN}}=\mathrm{HBinF}-\mathrm{CBF}$$

Q_23_ is the collateral flow entering the IJV between J2 and J3:

(5) $$Q_{23}=Q_{J2}-Q_{J3}$$

The above definition also applies to Q_12_:

(6) $$Q_{12}=Q_{J1}-Q_{J2}$$

For both Q_23_ and Q_12_, we define a positive flow when it has a direction from a collateral towards the jugular. Q_C-P_ (Collateral Proximal) is the collateral outflow which goes directly into the caval system:

(7) $$Q_{C-P}=\mathrm{HBinF}-\mathrm{HBoutF}$$

Finally, all the flows defined above satisfy the continuity equation:

(8) $$\left(Q_{C-D}+Q_{\mathrm{FN}}\right)-\left(Q_{23}+Q_{12}+Q_{C-P}\right)=0$$

#### *Calculated indexes*

The above measured haemodynamic parameters also allow to extrapolate four indexes^a^:

1. Delta Cerebral Venous Outflow (DCVO), defined as:

(9) $$\begin{array}{lll} \mathrm{DCVO} & = & \left(\frac{Q_{J1s}+Q_{\mathrm{VVs}}}{\mathrm{HBinF}}{|}_{\mathrm{Supine}}-\frac{Q_{J1s}+Q_{\mathrm{VVs}}}{\mathrm{HBinF}}{|}_{\mathrm{Upright}}\right)\\ & & \times 100 \end{array}$$

This index represents the normalized outflow difference between the supine and the upright position, as measured at the J1 level.

2. Distal Jugular and Vertebral Draining Index (DJVDI), defined as:

(10) $$\mathrm{DJVDI}=\frac{\mathrm{CVO}}{\mathrm{HBinF}}\times 100$$

This index represents the percentage of the blood entering in the head that is drained directly from the IJVs at level J3 and from the VVs.

3. Collateral Flow Index (CFI), defined as:

(11) $$\mathrm{CFI}=\frac{Q_{C-P}}{\mathrm{HBinF}}\times 100$$

This index represents the percentage of the blood entering in the head that is drained from collateral vessel instead to be drained from the IJVs or from the VVs.

4. Cerebral Collateral Draining Index (CCDI), defined as:

(12) $$\mathrm{CCDI}=\frac{Q_{C-D}}{\mathrm{CBF}}\times 100$$

This index represents the percentage of the blood entering the brain that is drained from collateral vessels instead to be drained from the IJVs or from the VVs. The suffix ′s′ in VVs, J1s and J3s indicates that both left and right flow are considered.

### Phase two of the study

We tested our model on a second population represented by ten patients (age ranging from 37 to 45 y.o., male:female ratio 5:5) affected by CCSVI. Such patients have been screened by the same ECD criteria among those affected by multiple sclerosis. All the selected patients showed a positivity of criterion 3, (i.e. presence of documented intra-luminal obstacles such as septa, membranes, webs, etc.) [5].

### Statistical analysis

Data are expressed as mean ± sd. The haemodynamic parameters were analyzed either separately in the different jugular sides, or as a whole. Differences among the parameters assessed in both healthy volunteers and in CCSVI patients were tested by means of Wilcoxon-Mann–Whitney U-test; p value < 0.05 was considered significant.

#### *Informed consent*

The entire cohort of investigate subjects was informed about the methods and purpose of the experimental procedure and agreed to participate by signing an informed consent form. This study was in accordance with the Ethical Standards of the Committee on Human Experimentation of the University of Ferrara.

## Results

### First phase of the study

#### *Arterial inflow*

The control subjects were successfully investigated. Calculated HBinF was 956 ± 105 ml/min, subdivided in 843 ± 200 ml/min in the CCAs, 462 ± 90 ml/min in the ICs, 255 ± 59 ml/min in the ECAs and 176 ± 72 ml/min in the VAs. Such values are similar to what was previously reported [17].

#### *Venous outflow indexes*

The calculated DCVO, DJDVI, CFI and CCDI indexes values are reported in Table 1 with their standard deviation and their meaning are discussed throughout the following text.

**Table 1:** Mean value (v) and standard deviation (sd) for Delta Cerebral Venous Outflow (DCDVI), Distal Jugular and Vertebral Draining Index (DJVDI), Collateral FLow Index (CFI) and Cerebral Collateral Draining Index (CCDI)

			**DCVO**	**DJDVI**	**CFI**	**CCDI**
**Supine**	**Controls**	v	5	45	1	33
		sd	10	17	3	24
	**Patients**	v	-42	33	61	53
		sd	82	17	27	23
**Upright**	**Controls**	v		41	9	39
		sd		10	19	16
	**Patients**	v		41	33	40
		sd		24	31	31

#### *Venous outflow in supine posture*

In Table 2 we report CSA, major axis, TAV, and Q respectively for right and left IJV, measured in supine; they increase from J3 to J1, and in J2 these values correspond to what was previously reported [18]. TAV increases significantly from J2 to J1, leading of course to increased Q values. The Q measured in left and right J2 plus VVs is about 11% less than the value reported by Doepp et al. [4]. However, this is coherent with a 14% higher CBF measured by the same authors in their normal subjects.

**Table 2:** Mean values (v) and standard deviation (sd) for cross sectional area (CSA), Major axis, TAV and flow (Q) in Jugular and Vertebral veins

				**CSA [cm**^**2**^**]**		**Major axis [cm]**		**TAV [cm/s]**		**Q [ml/min]**
			**right**	**left**	**right**	**left**	**right**	**left**	**right**	**left**
** J3**										
**Supine**	**Controls**	**v**	0,26	0,21	0,79	0,74	25,28	25,32	190,72	167,41
		**sd**	0,14	0,11	0,27	0,25	9,16	11,80	126,74	93,25
	**Patients**	**v**	0,29	0,18	0,85	0,68	17,33	18,08	139,29	77,73
		**sd**	0,19	0,15	0,21	0,28	10,78	22,06	108,64	80,03
**Upright**	**Controls**	**v**	0,12	0,13	0,57	0,56	36,55	23,75	153,03	94,43
		**sd**	0,10	0,11	0,32	0,29	21,10	16,98	97,15	73,91
	**Patients**	**v**	0,18	0,12	0,73	0,52	41,85	23,51	201,98	96,09
		**sd**	0,04	0,09	0,07	0,22	38,82	41,22	171,83	141,91
** J2**										
**Supine**	**Controls**	**v**	0,37	0,28	1,09	0,94	27,54	47,91	238,94	293,36
		**sd**	0,31	0,19	0,30	0,22	18,99	25,43	148,08	140,17
	**Patients**	**v**	0,28	0,25	0,96	0,91	34,32	44,07	273,12	219,73
		**sd**	0,17	0,13	0,74	0,25	22,61	33,73	245,33	190,40
**Upright**	**Controls**	**v**	0,11	0,07	0,64	0,56	43,61	55,64	151,04	162,55
		**sd**	0,04	0,04	0,30	0,33	30,02	28,36	119,50	156,05
	**Patients**	**v**	0,12	0,06	1,16	0,75	40,64	38,91	261,10	126,66
		**sd**	0,09	0,04	0,52	0,30	50,23	52,70	247,47	102,74
** J1**										
**Supine**	**Controls**	**v**	0,48	0,50	1,24	1,09	51,69	51,20	712,56	606,27
		**sd**	0,30	0,14	0,34	0,39	33,48	45,54	451,21	417,79
	**Patients**	**v**	0,45	0,36	1,32	0,63	15,40	16,10	151,61	117,87
		**sd**	0,18	0,22	0,33	0,60	19,63	16,94	238,72	121,70
**Upright**	**Controls**	**v**	0,16	0,17	0,83	0,87	112,12	86,31	755,16	469,62
		**sd**	0,16	0,11	0,33	0,36	47,99	42,14	690,26	287,23
	**Patients**	**v**	0,18	0,16	0,74	0,73	63,30	42,00	439,98	334,23
		**sd**	0,10	0,09	0,16	0,26	61,73	49,20	319,58	343,94
** VV**										
**Supine**	**Controls**	**v**	0,05	0,04			30,44	22,33	42,16	24,38
		**sd**	0,03	0,02			27,73	14,20	40,42	10,66
	**Patients**	**v**	0,05	0,04			21,56	23,15	49,37	39,70
		**sd**	0,04	0,03			21,81	25,46	72,27	53,63
**Upright**	**Controls**	**v**	0,04	0,05			52,72	50,69	53,72	81,33
		**sd**	0,02	0,03			28,95	26,30	27,18	67,13
	**Patients**	**v**	0,03	0,03			22,07	16,93	68,58	58,65
		**sd**	0,04	0,06			35,47	26,57	108,05	84,65

In our sample, the rate of HBinF drained by the IJVs is 37% in J3, 55% in J2 and more than 90% in J1, respectively, and thus suggesting a re-entry of significant blood volume along the jugular vein through the collaterals.

It is worth noting that more than 90% of HBinF is drained by the IJVs in upright posture. Although there is evidence in the literature that VVs are the main draining route in this position, our finding refers to measurements in J1, a segment not previously investigated. Since this is a preliminary study that refers to a small sample size, it is important to investigate the current finding so as to determine the exact role of the gravitational gradient [1,2] in the distribution changes of venous outflow from the brain.

In addition, our model permits to derive the volume of blood flowing into the collaterals of normal subjects, through the methodology reported above. As shown in Table 2, this is a consistent amount of blood never measured before: up to 350 ml/min for the collaterals entering between J2 and J3 and more than 500 ml/min for the collaterals entering between J1 and J2. However, the mean measured CFI was 1 ± 3%, clearly indicating that a very little fraction of blood flowing along the collaterals of normal subject bypasses the IJV and re-enters directly into the caval system.

The index DJDVI and CCDI were respectively 45 ± 17% and 33 ± 24%. The DJDVI reveals that for healthy controls in upright position, 45% of the mean HBinf is drained both by the IJVs at the J3 level and the VVs. Concerning the CCDI index, we found that about 33% of the CBF is drained through the collaterals. However, since the CFI is only 1%, this blood always flow into the jugulars.

#### *Venous outflow in upright posture*

In Table 2 we report CSA, major axis, TAV, and Q respectively for left and right IJV as measured in upright; TAV increases from J3 to J1, whereas CSA and major axis are apparently constant.

In our sample the rate of HBinF drained by the IJVs is 26% in J3, 33% in J2 and more than 90% in J1 and thus suggesting, also in upright, a re-entry of significant volume of blood along the jugular vein through the collaterals. As previously reported [3], we measured a significant reduction of the sum of the jugular and vertebral outflow in J2 when comparing the sitting with the supine position (mean 448 ml/min vs 600 ml/min).

Finally, the index DJDVI and CCDI were respectively 41 ± 10% and 39 ± 16%, while DCVO value was 5 ± 10%. In this case the DJDVI reveals that for healthy controls in upright position, 41% of the mean HBinf is drained both by the IJVs at the J3 level and the VVs. Concerning the CCDI index, we found that about 40% of the CBF is drained through the collaterals.

### Second phase of the study

#### Arterial inflow

All the patients were successfully investigated. Calculated HBinF was 908 ± 90 ml/min subdivided in 758 ± 138 ml/min in the CCAs, 444 ± 123 ml/min in the ICs, 230 ± 83 ml/min in the ECAs and 192 ± 60 ml/min in the VAs.

#### Venous outflow in supine posture

In Table 2 we report CSA, major axis, TAV, and Q respectively for left and right IJV, measured in supine. Differently from what we measured in control subjects, Q and TAV increased from J3 to J2 but not from J2 to J1. This is confirmed by the rate of the HBinF drained in the different segments of the IJV, respectively 24% in J3 and 54% in J2, but dramatically reduced to 32% in J1. Since CFI is 61 ± 27%, our model permits to discover a significant volume of blood flowing in the collateral network rather than in the terminal segment of the IJV.

Finally, the index DJVDI and CCDI were respectively 33 ± 17% and 53 ± 23%. The high CCDI value shows that a significant fraction of the CBF is drained by the collaterals rather than the main routes (IJV e VV).

#### Venous outflow in upright posture

In Table 2 we report CSA, major axis, TAV, and Q respectively for left and right IJV, measured in sitting; TAV and Q increases from J3 to J1, whereas CSA and major axis are apparently constant. The Q measured in left and right J2 plus VVs is about 510 ml/min.

The rate of HBinF drained in the different segments of the IJV is 32% in J3, 41% in J2 and more than 80% in J1. Besides, we found a consistent amount of blood, more than 500 ml/min, for both the collaterals entering between J2 and J3 and the collaterals entering between J1 and J2. The CFI is 33 ± 31%, so considerably lower than the supine position and thus indicating a reduction of blood circulating into the collateral network when the drainage occurs in favour of gravity.

Finally, the index DCVO DJDVI and CCDI were -42 ± 80%, 41 ± 24%, and 40 ± 31%, respectively.

#### Comparison between healthy controls and CCSVI patients according to the model

The HC cohort was compared to the CCSVI one. It is worth noting that HBinF and CBF did not show significant differences among the groups (p > 0.14 and p > 0.95 respectively), hence, permitting a more focused comparison of the differences of cerebral venous return between the two groups. From this point of view, the main difference is the flow in J1 which, for the CCSVI patients, is about 70% less than the healthy controls (p < 0.001). Consequently, in the latter we found a significant higher CFI (p < 0.0002), clearly indicating the level of activation of the collateral network in the latter group.

#### *Comparison in supine position*

The above results are the consequence of the significant flow differences measured between the two groups in the supine position. While in J3 the flow Q showed simply a trend (p = 0.07), in J1 both Q and the CFI dramatically decreased (p < 0.000002). The latter result depends on the fact that the CFI index for healthy controls is separated by two standard deviations from the CFI of the MS patients (see Table 1).

#### *Comparison in upright position*

By turning the subjects in sitting posture, we did not find out significant differences in the control group by comparing the flow in the two postures. The major limitation is linked with the small sample and the big sd. To the contrary, by turning the CCSVI patients from supine to upright there is a drop in the the jugular flow in J1.

## Discussion

### First phase of the study

In the first part of the study we tested the model on a HC cohort based on medical history and a controversial US CCSVI screening [5,19-27]. However, a recent meta-analysis clearly shows that the majority of HC are not affected by CCSVI [28]. Finally, also MRI data, more objective and less operator dependent with respect to US, are still controversial because there are confirmatory and not confirmatory studies [25-27,29].

Our measurements of the inflow are definitively comparable with previously published data [17]. Same result was found for the evaluation of the outflow, because the Q value assessed in J3, J2 and in the VVs are similar to the values previously reported [3,4,19].

The novelty of the present study is the application of a complete model which takes into account the haemodynamics of cerebral venous return normalized to the HBinF. Our model, for the first time, also includes J1 and haemodynamic analysis of collaterals.

Furthermore, we confirm that the flow in the IJV increases from the jaw to the chest [6], with consequent increased rate of the initial HBinF which is drained by the three considered segments. This is likely due to the re-entry of the collaterals into the main outflow route, as demonstrated by the calculated part flowing in the collateral network. However, we underline that in HC only 1% of the HBinF was not measured in the final amount of the HBoutF, thus indicating that a very small amount of blood volume in physiology re-enters through the collaterals into the caval system by skipping the IJV.

However, even if our model is complete and permits to better detail the modality of drainage from the brain, we would discuss some potential shortcomings linked to the proposed experimental setting. The first observation is that, due to the work of the valve leaflets, in J1, also in physiologic condition, the flow is turbulent. Flow turbulences may potentially affect the measurements of the TAV, so resulting in an overestimated assessment of Q in J1 [30]. This means that the assessment is less precise with respect to a straight venous segment having an ideal laminar flow and this issue will be subject of future work.

A second limitation in the ECD assessment of Q in J1 is linked to the technical feasibility to place a steering angle different from 0° in the lumen, especially when insonating the supra-clavicular fossa in subjects with more pronounced clavicular bone [31].

Moreover, we estimated an uncertainty of about 5 degrees in ϵ when the operator places the sample volume into the J1 lumen. This uncertainty has been estimated by asking the operator to recursively assess the correct Doppler angle so as to evaluate the standard deviation of the mean. The variability of such technical aspect may potentially lead to an overestimation of the TAV, finally affecting the Q up to 20% [16].

Finally, statistical comparisons were not adjusted for demographics and vascular risk factors, but this analysis is beyond the aim of the present study where we tested the feasibility of the proposed model in a limited sample size.

### Second phase of the study

Once we developed the above described model, we tested its potential utility in the clinical setting by performing preliminary measurements in CCSVI condition. The main finding of the second phase is the significantly higher fraction of blood flowing in the collateral network of the CCSVI patient with respect to the HC. Our model permits to extrapolate that about 60% of the initial HBinF is transported directly to the caval system, significantly higher than 1% of CFI assessed in HC. This quantity dramatically increases because does not include only the flow drained in the soft tissue of the face and neck, but likely a high rate of blood transported by the IJV. Such a vision is clearly supported by two measured parameters. The former is the decreased Q passing from J2 to J1 (about 40%). The latter is the negative flow measured in C1-2 in the same population, which indicates the inversion of flow direction in the upper collaterals and it is likely due to the increased resistance exhibited by the terminal jugular vein. This anomalous behaviour could be the consequence of the intra-luminal obstacles detected in J1 at the time of ECD screening.

**Figure 2:**
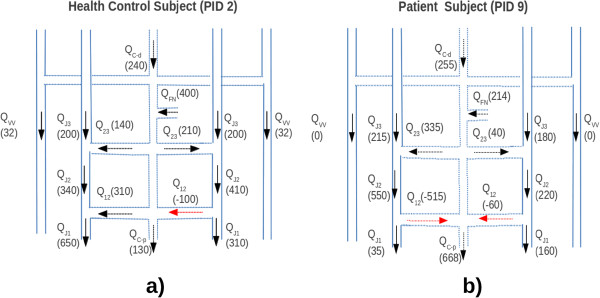
**Comparison between the neck pathway of a) HC subject and b) CCSVI subject.** The numbers in parenthesis refer to the blood flow in ml/min. The dashed arrows in red colour have an opposite direction.

As an example, we applied the proposed model to compare HC subjects with CCSVI ones having same age and gender. Comparing Figure 2a with Figure 2b it is apparent that the flow at J3 and J2 are comparable, as well as the amount of blood flowing in the collaterals.

What is dramatically changed is the value of Q in J1, where we assessed in the control subjects a further increase which leads to an overall amount of about 90% of the HBinF. On the contrary, a flow decrease of about 50% is apparent when passing from J2 to J1 in the CCSVI case. Finally, also the application of the model in venous pathology clearly shows the same limitations described above.

#### Comparison in supine position

We proposed four novel parameters in order to characterize the cerebral venous return, but only the CFI showed significant differences between the two cohorts in our study. CFI expresses the blood flowing into collateral network rather than the main outflow routes. It is really interesting that CCDI, which represents the rate of the inflow going into the collaterals at J3 level, is not significantly different in the two cohorts. This result may be linked to the limited flow assessed in the IJV at J1 level.

#### Comparison in upright position

The main finding is represented by the considerable drop of IJV flow measured in J1 when changing the position of MS patients, as also previously assessed by Doepp [19] and Monti [21].

## Conclusion

We developed a new model that permits a detailed ECD quantification of the cerebral venous return, including an estimation of the amount of blood flowing from the collaterals to the caval system or to the IJV. The preliminary application of the model seems to indicate how a significant rate of the head inflow is drained by the collateral network rather than by the IJV in the CCSVI condition. This may help the interpretation of several findings assessed with different techniques, where it was not possible to assess the outflow contribution of the collateral network, as well as the rate of the inflow going in the main venous paths. For instance, the higher flow in the collateral network may explain the longer cerebral circulation time measured by means of contrast-enhanced US, as well as the slower discharge and increased resistance measured in MS [32-34]. Our preliminary report needs to be further corroborated by reproducibility analysis, wider number of subjects and pathological conditions, and possibly, by a multicenter design. This may lead to a further advancement for the circulatory quantification of the CCSVI condition in the clinical setting via ultrasonography.

## Endnote

^a^The named indexes are subject to copyright.

## Abbreviations

Brain-C: Brain compartment; CBF: cerebral blood flow; CCA: Common carotid artery; CFI: Collateral Flow Index; CSA: Cross sectional area; CCDI: Cerebral Collateral Draining Index; CCSVI: Chronic Cerebrospinal venous insufficiency; CVO: Cerebral venous outflow; DCVO: Delta Cerebral Venous Outflow; DJVDI: Distal, Jugular Vertebral Draining Index; ECA: External carotid artery; ECD: Echo coulor Doppler; HBinF: Head blood in-flow; HBoutF: Head blood out-flow; HC: Healthy control; ICA: Internal carotid; IJVs: Internal jugular veins; PT: Total of patients; FN-C: Facial and neck compartment; SV: Sample volume; TAV: Time average velocity; VA: Vertebral artery; VVs: Vertebral veins.

## Competing interests

The authors declare that they have no competing interests.

## Authors’ contributions

PZ conceived the study, contributes to develop the model and wrote the paper. FS developed the model and the equations, collected and analyzed the raw data, performed the statistical analysis and wrote the paper. EM performed the Echo Colour Doppler screening and collected the data. AT wrote the paper and revised it critically. AMM and SM contribute to collect and analyze the data. MG provided scientific supervision and founded the study. All authors participated in the design study. All authors read and approved the final manuscript.

## Pre-publication history

The pre-publication history for this paper can be accessed here:

http://www.biomedcentral.com/1471-2377/13/81/prepub
